# Clinical and Radiographic Outcomes of Customized Titanium Mesh vs. Screw Tent‐Pole Grafting: A Retrospective Study

**DOI:** 10.1111/cid.70139

**Published:** 2026-03-20

**Authors:** G. Wurtz, F. Bagnasco, M. Menini, P. Pesce, D. Baldi, N. De Angelis

**Affiliations:** ^1^ Department of Surgical Sciences and Integrated Diagnostics University of Genoa DISC Genova Italy

**Keywords:** alveolar ridge augmentation, bone substitutes, computer‐aided design, cone‐beam computed tomography, dental implants, guided bone regeneration, titanium mesh

## Abstract

**Background:**

Guided bone regeneration (GBR) is a predictable approach for managing severe alveolar ridge deficiencies prior to implant placement. Resorbable collagen membranes supported by tenting screws are widely used, although space maintenance in non‐contained defects may be challenging. Customized CAD/CAM titanium meshes have been introduced to enhance graft stability and surgical workflow, but comparative clinical evidence remains limited.

**Objective:**

To compare clinical, radiographic, procedural, and peri‐implant outcomes of customized CAD/CAM titanium meshes versus resorbable collagen membranes supported by tenting screws for horizontal and/or vertical alveolar ridge augmentation.

**Materials and Methods:**

This retrospective study included 40 patients with severe alveolar ridge defects, allocated to two groups (*n* = 20 each). Both groups received particulate bone grafts stabilized either with tenting screws and a resorbable collagen membrane or with a patient‐specific CAD/CAM titanium mesh. Cone‐beam computed tomography (CBCT) scans at baseline and 6 months were used to assess vertical and horizontal bone gain. Intraoperative time, complications, pseudo‐periosteum formation, implant survival, and peri‐implant marginal bone levels at prosthetic loading and at 5‐year follow‐up were recorded.

**Results:**

At 6 months, mean bone height reached 8.7–8.93 mm in the maxilla and 9.25–9.35 mm in the mandible, while mean ridge width ranged from 4.7 to 5.3 mm, with no significant intergroup differences (*p* > 0.05). Mean peri‐implant marginal bone loss was limited and remained stable from prosthetic loading to the 5‐year follow‐up in both groups. Mean operative time was significantly shorter in the customized mesh group, 72.7 min (range: 60–85) for the Tent‐pole group and 62.4 min (range: 60–65) for the Ti‐mesh group. All 60 implants placed in 40 augmented sites survived, with no implant failures and no need for additional grafting procedures.

**Conclusions:**

Both GBR techniques provided comparable bone regeneration and long‐term peri‐implant stability, while customized CAD/CAM titanium meshes were associated with reduced operative time.

## 1 Introduction

The alveolar ridge, which supports the dentition, undergoes pronounced dimensional changes following tooth loss [1]. Even after uneventful socket healing, significant buccal and vertical resorption typically occurs within the first 6–12 months, resulting in a net reduction of ridge volume [2, 3]. As a consequence, residual horizontal and vertical defects are frequently observed, and spontaneous recovery of the original ridge anatomy is rare [4]. These volumetric alterations may compromise optimal implant positioning, prosthetic rehabilitation, and esthetic outcomes, often necessitating adjunctive bone augmentation procedures [5, 6].

Guided bone regeneration (GBR) represents a well‐established approach for reconstructing deficient alveolar ridges [7]. Tent‐pole technique protocols generally combine particulate bone grafts with barrier membranes, most commonly resorbable collagen membranes or non‐resorbable polytetrafluoroethylene (PTFE) [8, 9, 10, 11, 12] However, both membrane types present inherent limitations. Resorbable membranes lack sufficient mechanical stability and are prone to collapse under soft‐tissue pressure, requiring additional space‐maintaining devices such as tenting screws or graft material [3]. In contrast, non‐resorbable PTFE membranes provide greater rigidity but are associated with an increased risk of early exposure and infection, often necessitating premature removal and potentially compromising regenerative outcomes [13].

A systematic review by Pourdanesh et al. [14] analyzed the clinical outcomes of dental implants placed in sites augmented using the tenting approach, reporting favorable bone regeneration and implant survival rates comparable to other established GBR techniques. The authors highlighted that mechanical support of the barrier is a key determinant of regenerative success, regardless of the membrane material used. These findings support the concept that space maintenance, rather than the barrier itself, plays a central role in GBR outcomes.

Titanium meshes were introduced to improve space maintenance and graft stability in GBR procedures [15]. Owing to their structure and favorable mechanical properties, titanium meshes effectively resist soft‐tissue compression and preserve the intended regenerative volume throughout healing [16, 17]. In addition, titanium exhibits excellent biocompatibility and corrosion resistance, while its porous architecture supports osteoconduction and vascularization. Clinically, titanium meshes have demonstrated lower exposure rates than PTFE barriers, and when exposure occurs, soft‐tissue integration may allow healing to proceed without mandatory mesh removal [15, 16, 18]. These characteristics make titanium meshes particularly suitable for severe ridge atrophy and non‐contained defects.

Recent developments in digital planning and manufacturing have enabled the production of customized, CAD/CAM‐fabricated titanium meshes [19, 20]. Using cone‐beam computed tomography (CBCT) data, patient‐specific meshes can be designed to conform precisely to the planned ridge morphology and subsequently manufactured via additive techniques [21]. This digital workflow allows for passive adaptation of the mesh, minimizes intraoperative manipulation, and reduces surgical time. Moreover, accurate preoperative planning may improve graft stability, facilitate flap closure, and decrease soft‐tissue tension [22, 23].

Despite these advances, the optimal GBR approach remains controversial. Evidence supporting the clinical superiority of customized titanium meshes over conventional membrane‐based GBR protocols is still limited. To date, only a small number of studies have directly compared patient‐specific titanium meshes with standard GBR techniques, and data on dimensional bone gain and complication rates remain inconclusive [16, 24].

The aim of this retrospective study was to compare the clinical outcomes of guided bone regeneration performed using customized CAD/CAM titanium meshes with those achieved using a Tent‐pole technique protocol. Maxillary and mandibular sites treated with patient‐specific titanium meshes in combination with particulate bone grafts were compared with sites augmented using resorbable collagen membranes supported by tenting screws. Dimensional bone gain, graft stability, complication rates, and peri‐implant marginal bone levels at the time of prosthetic loading and at 5‐year follow‐up were evaluated to assess also the stability of the peri‐implant newly regenerated bone.

## 2 Materials and Methods

### 2.1 Sample Size Calculation

This is a retrospective observational study in which patients were allocated to groups based on the surgical treatment they received in routine clinical practice, rather than by predefined exposure or random assignment. Group classification was performed post hoc according to the surgical approach applied. The study compared two guided bone regeneration (GBR) techniques, with regenerated linear bone gain as the primary outcome measure. A total of 40 patients were included, with 20 subjects allocated to each treatment group. Owing to the retrospective design and the limited number of eligible cases available in the clinical records, an a priori sample size calculation was not feasible.

A post hoc power analysis was therefore performed to evaluate the statistical adequacy of the sample. The analysis was conducted using G*Power software (version 3.1; Heinrich Heine University, Düsseldorf, Germany), applying a two‐tailed independent‐samples *t*‐test with a significance level set at *α* = 0.05 and a target statistical power of 80% (1 − *β* = 0.80). Based on the available sample size (*n* = 20 per group), the minimum detectable standardized effect size was calculated.

**FIGURE 1:**
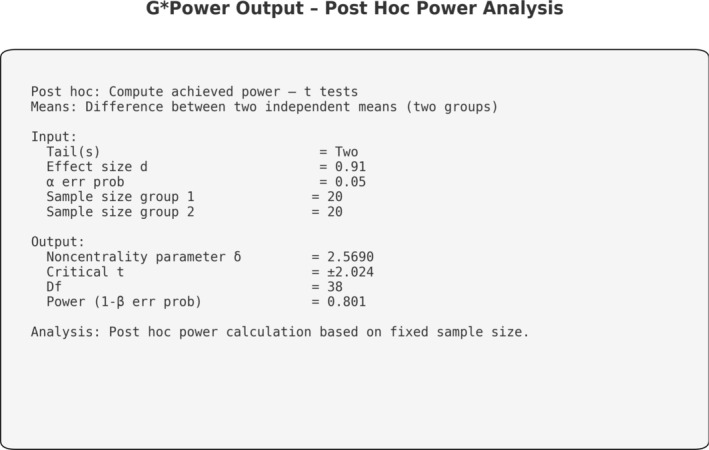
Sample size calculation parameters.

Under these assumptions, the study was adequately powered to detect an effect size of Cohen's *d* = 0.91, corresponding to a large effect. Given the absence of preliminary variance estimates and the retrospective nature of the investigation, this approach was considered appropriate and is consistent with previously published GBR studies using similar methodological frameworks [25, 26] (Figure 1).

### 2.2 Study Design and Ethical Considerations

This retrospective observational study was conducted in accordance with the principles of the Declaration of Helsinki and was approved by the local Ethics Committee (CERA; approval no. 2025/101). The study was reported in compliance with the Strengthening the Reporting of Observational Studies in Epidemiology (STROBE) guidelines [27].

Clinical records were retrieved from July to November 2025 from a private dental practice (Studio De Angelis Acqui Terme Italy) where all treatments had been performed in 2020. Written informed consent had been obtained from all patients for participation in the study, as well as separate authorization for the use of clinical images and radiographic data for research and publication purposes.

### 2.3 Eligibility Criteria

Patients were included in the analysis if they met the following criteria:

- treatment with guided bone regeneration (GBR) for vertical and/or horizontal alveolar ridge defects;
- absence of general contraindications to oral surgery.
- presence of initial 3D Cone Beam Computed Tomography

Exclusion criteria were: systemic conditions potentially affecting bone healing or surgical outcomes (including immunosuppression or immunocompromise, history of head and neck irradiation, uncontrolled diabetes, pregnancy or lactation); untreated periodontitis or inadequate oral hygiene and compliance; substance abuse; heavy smoking (> 10 cigarettes/day); psychiatric disorders or unrealistic treatment expectations; acute infection at the intended implant site; current or previous treatment with intravenous amino‐bisphosphonates; and participation in other clinical trials that could interfere with the present protocol.

*Reviewer‐oriented clarification:* exclusion criteria were defined a priori to minimize confounding factors known to influence GBR outcomes and wound healing, thereby improving internal validity despite the retrospective design.

### 2.4 Surgical Protocols

Patients were allocated into two groups according to the GBR technique performed.

#### 2.4.1 Group A: Tent‐Pole Technique

Deproteinized bovine bone mineral mixed with 50% autologous bone was used as graft material and covered with a resorbable collagen membrane stabilized by tenting screws.

#### 2.4.2 Group B: Customized Titanium Mesh GBR

Deproteinized bovine bone mineral mixed with 50% autologous bone was covered with a patient‐specific CAD/CAM titanium mesh, which was in turn overlaid with a resorbable collagen membrane.

All surgical procedures were performed following standardized protocols. In both groups, flap elevation, graft placement, barrier stabilization, and tension‐free wound closure were carried out according to established GBR principles.

*Reviewer‐oriented clarification:* the use of the same grafting material in both groups was intended to isolate the effect of the barrier system on regenerative outcomes.

### 2.5 Outcome Measures

Outcome measures were predefined and categorized as primary and secondary.

Primary outcomes included:

- dimensional bone gain, assessed as changes in ridge height and width between baseline (T0, preoperative) and 6 months post‐surgery (T1) by means of the measurements tolls included in the software IRYS (https://www.myray.it/en/radiology‐software/irys);
- incidence of surgical and postoperative complications.

Secondary outcomes included:

- presence and extent of pseudo‐periosteum formation at re‐entry, assessed during flap reopening and categorized according to previously described criteria [28];
- patient‐reported experience, assessed using a structured anonymous questionnaire administered immediately after surgery by a non clinical operator and returned 7 days postoperatively;
- intraoperative time, defined as the interval from the initial incision to placement of the final suture.
- marginal bone level around the implants at the loading time and 5 years after loading.

**FIGURE 2:**
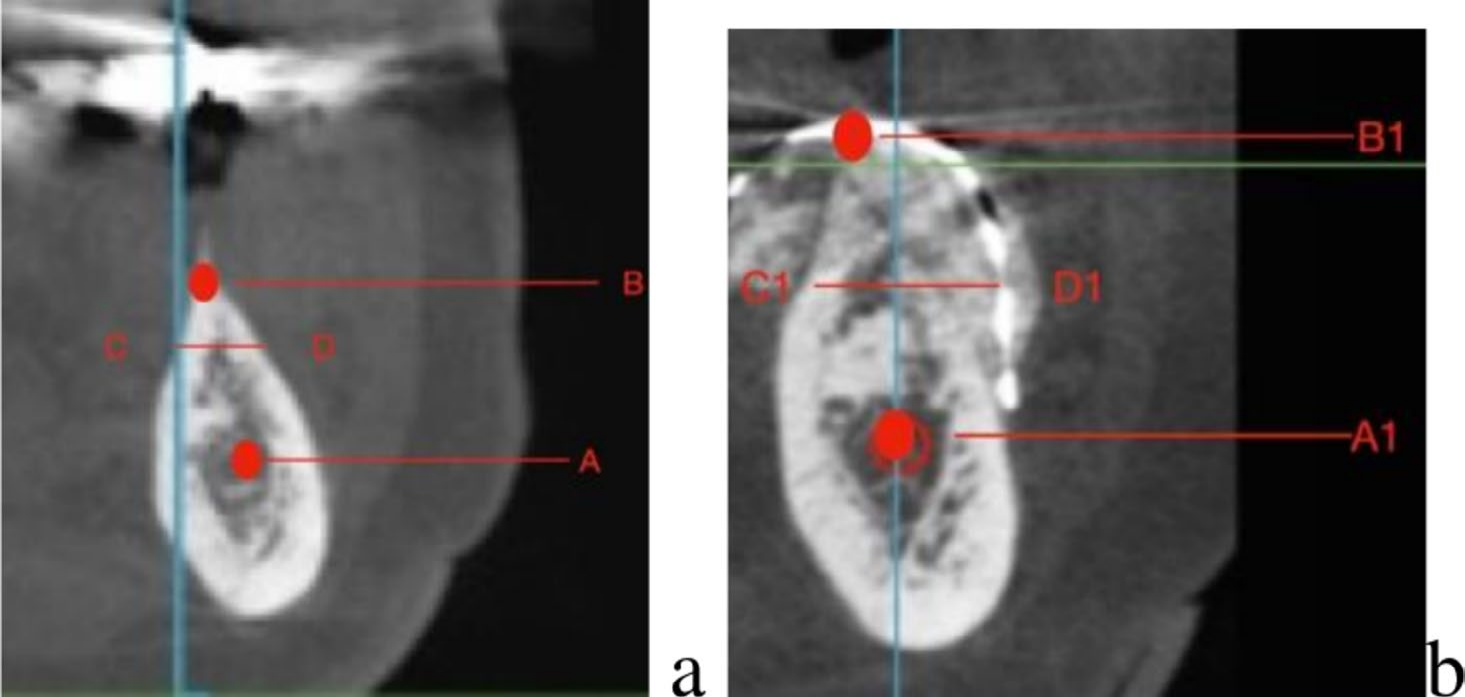
(a) Preoperative CBCT sagittal cut. (b) postoperative CBCT sagittal cut. Landmarks A and A1 identify the position of the alveolar canal, while B and B1 the most coronal point of the crestal height. The distance A → B and A1 → B1 are the preoperative and postoperative heights of the defect. Similarly landmarks C and C1 identify the lingual point used for the measurement of the mandibular or maxillary width, while D and D1 the buccal point. Therefore the distance C → D and C1 → D1 are the preoperative and postoperative widths of the defects.

Data were retrospectively collected from standardized clinical and radiographic records. The following variables were recorded for each patient: demographic data (age and sex); anatomical site (maxilla or mandible); defect characteristics (horizontal and/or vertical component); surgical technique (Tent‐pole or CAD/CAM Ti‐mesh) as displayed in Figure 2; operative time (defined as the interval from initial incision to final suture); radiographic measurements of ridge dimensions at baseline (T0) and at re‐entry (T1), including ridge height and width; vertical and horizontal bone gain; presence and thickness of pseudo‐periosteum at re‐entry; implant placement data; marginal bone level changes at 5‐year follow‐up; and biological complications, including infection, exposure, or graft failure. All measurements were obtained using standardized radiographic protocols and clinical records.

### 2.6 Surgical Procedure—Group A (Tent‐Pole Technique)

**FIGURE 3:**
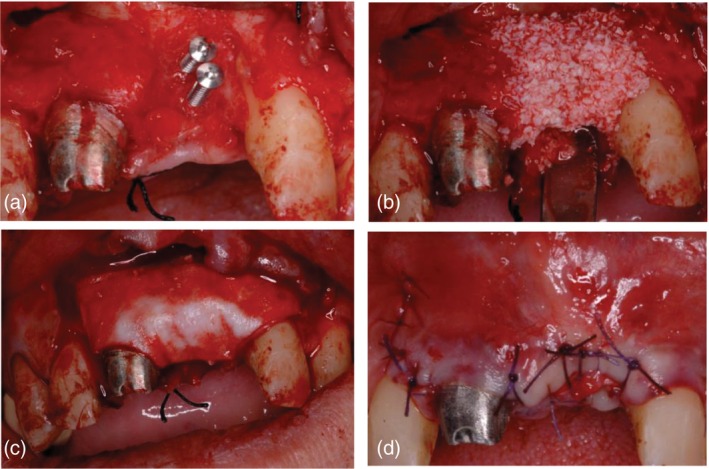
Case of Tent‐pole Group (A) (a) Defect debrided and tenting screws in place, (b) mixture of autogenous bone and xenograft, (c) double layer collagen membrane, (d) suture.

For Tent‐pole group (Figure 3), following preoperative clinical examination and three‐dimensional radiographic assessment of the recipient site, standard disinfection protocols and local anesthesia were applied. A crestal incision was performed, and a full‐thickness mucoperiosteal flap was elevated to fully expose the defect area. Vertical releasing incisions were avoided whenever possible and were performed only when necessary to prevent flap tearing and to allow for tension‐free closure.

The recipient site was carefully debrided to remove fibrous tissue and non‐vital bone using hand curettes under copious saline irrigation. Autologous bone was subsequently harvested from the mandibular ramus using a bone scraper and temporarily stored in sterile saline solution. The harvested autologous bone was then mixed in a 1:1 ratio with a deproteinized bovine bone mineral to obtain the final grafting material.

**FIGURE 4:**
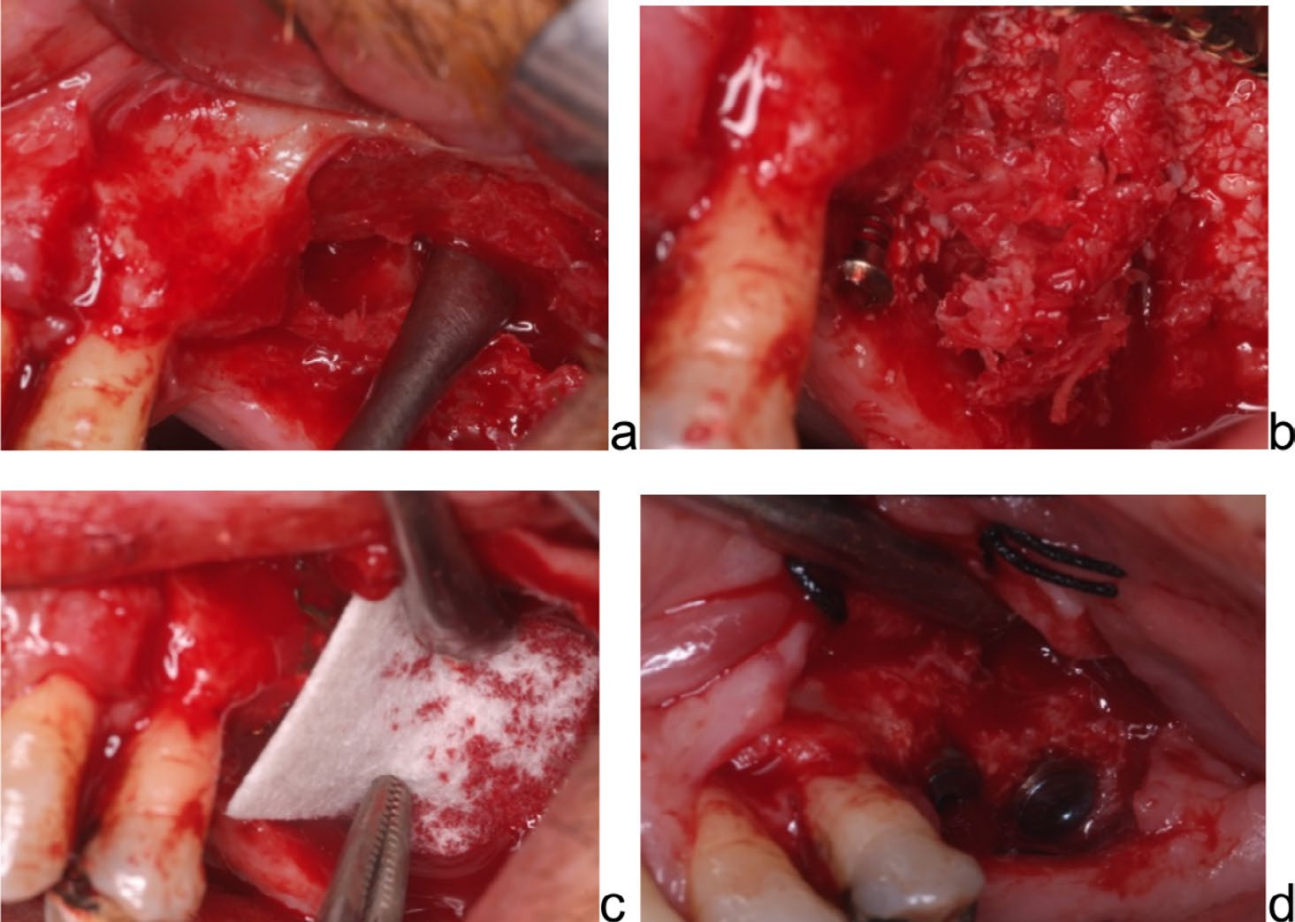
A case of maxillary vertical bone augmentation (a–d) shows the 7 months follow up and the implant placement in the regenerated area.

Titanium tenting screws were placed to maintain the regenerative space and prevent membrane collapse without exerting excessive pressure on the underlying tissues. The graft material was then gently packed into the defect, ensuring complete defect filling and contouring while avoiding excessive compression. Another case is displayed in Figure 4.

A resorbable collagen membrane was subsequently positioned to cover the grafted area. The membrane was carefully adapted to ensure complete coverage of the defect and stabilized with fixation pins, avoiding tension or displacement. Primary wound closure was achieved through passive flap advancement, facilitated by periosteal releasing incisions. Suturing was performed using a double‐layer technique, consisting of internal horizontal mattress sutures at the mucogingival junction and superficial interrupted sutures.

Postoperative follow‐up included clinical evaluations at 7 and 14 days after surgery. Radiographic assessment using three‐dimensional imaging was performed at 6 months postoperatively for all patients.

A standardized postoperative pharmacological regimen was prescribed for both groups. Antibiotic prophylaxis consisted of amoxicillin–clavulanic acid (1 g every 8 h) initiated 1 day prior to surgery and continued for a total of 6 days. Patients with a documented penicillin allergy received azithromycin (500 mg once daily), starting the day before surgery. Given the extent of flap elevation and bone exposure, systemic corticosteroids (betamethasone) were administered postoperatively (2 mg daily for the first 2 days, followed by 1 mg daily for the subsequent 3 days). Analgesic medication was prescribed on an as‐needed basis.

Plaque control was maintained using chlorhexidine mouth rinses (0.2%) four times daily for 14 days. Patients were instructed to avoid mechanical cleaning of the surgical site during the initial healing phase.

### 2.7 Surgical Procedure—Ti‐Mesh Group Group B (Customized Titanium Mesh GBR)

**FIGURE 5:**
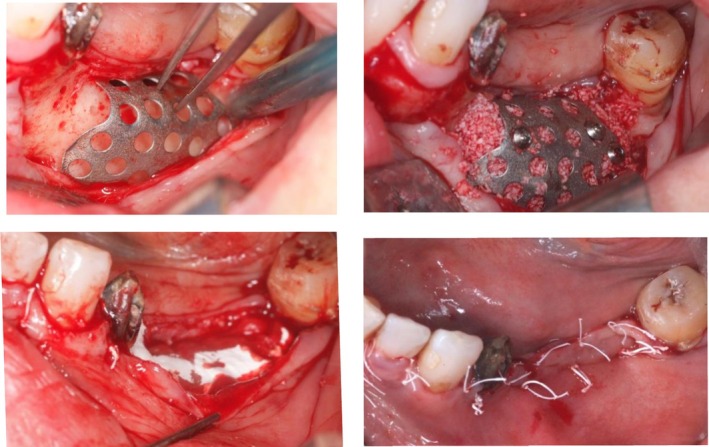
Ti‐mesh group: Customized Ti mesh try in; fixation of the mesh; a resorbable collagen membrane is placed onto the mesh to preserve the graft but mostly to prevent any early exposure; suture.

For Ti‐mesh group (Figure 5), following preoperative clinical examination and three‐dimensional radiographic assessment of the recipient site, standard disinfection protocols were applied and local anesthesia was administered. A crestal incision combined with vertical releasing incisions was performed to allow elevation of a full‐thickness mucoperiosteal flap. This approach provided adequate exposure of the bone defect and surrounding anatomical structures and enabled tension‐free coverage of the customized titanium mesh during wound closure.

The recipient site was prepared by thorough debridement, with removal of fibrous and granulation tissue using hand instruments under copious sterile saline irrigation.

Autologous bone was harvested from the mandibular ramus using bone scrapers and drills and temporarily stored in sterile saline solution. The harvested bone was then mixed in a 1:1 ratio with a deproteinized bovine bone mineral to obtain the grafting material.

Prior to graft placement, the prefabricated patient‐specific CAD/CAM titanium mesh was positioned to verify its fit and adaptation to the defect morphology. The bone graft mixture was subsequently placed into the defect and gently compacted to achieve stable volume reconstruction, avoiding excessive compression or overfilling.

A resorbable collagen membrane was subsequently positioned over the customized titanium mesh and adapted to ensure complete coverage of the grafted area. The membrane was intentionally extended beyond the margins of the mesh to promote soft‐tissue integration and enhance wound stability.

Primary wound closure was achieved through passive flap advancement, facilitated by periosteal releasing incisions. Suturing was performed using a double‐layer technique, consisting of internal horizontal mattress sutures at the level of the mucogingival junction and superficial interrupted sutures.

**FIGURE 6:**
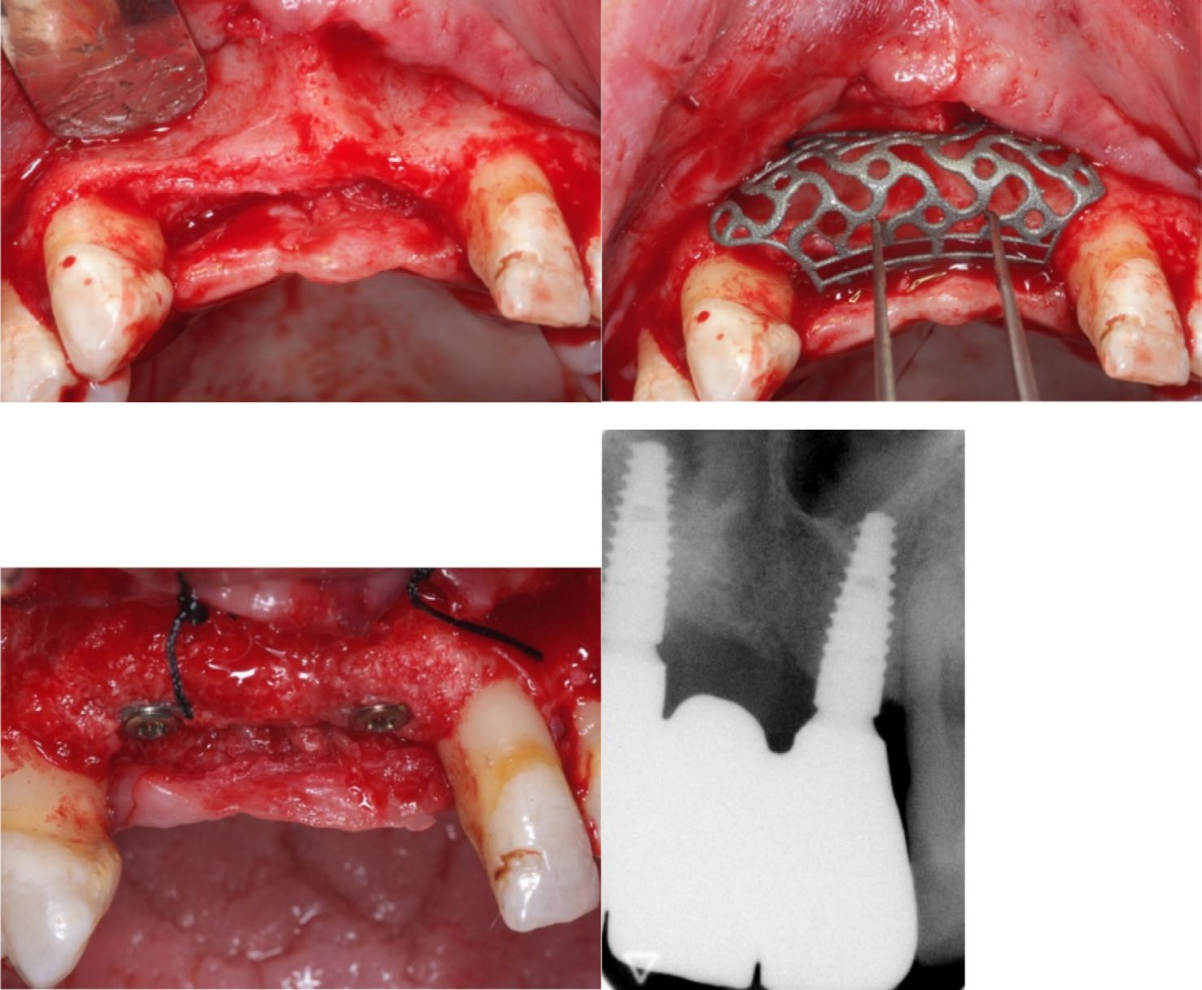
Vertical and horizontal defect of the maxillary anterior region; customized Ti mesh try in and results of the augmentation with the implants placement, which are also reported at the 5th year after the insertion.

Another case id displayed in Figure 6.

Postoperative follow‐up and pharmacological management were identical to those described for Tent‐pole group.

**FIGURE 7:**
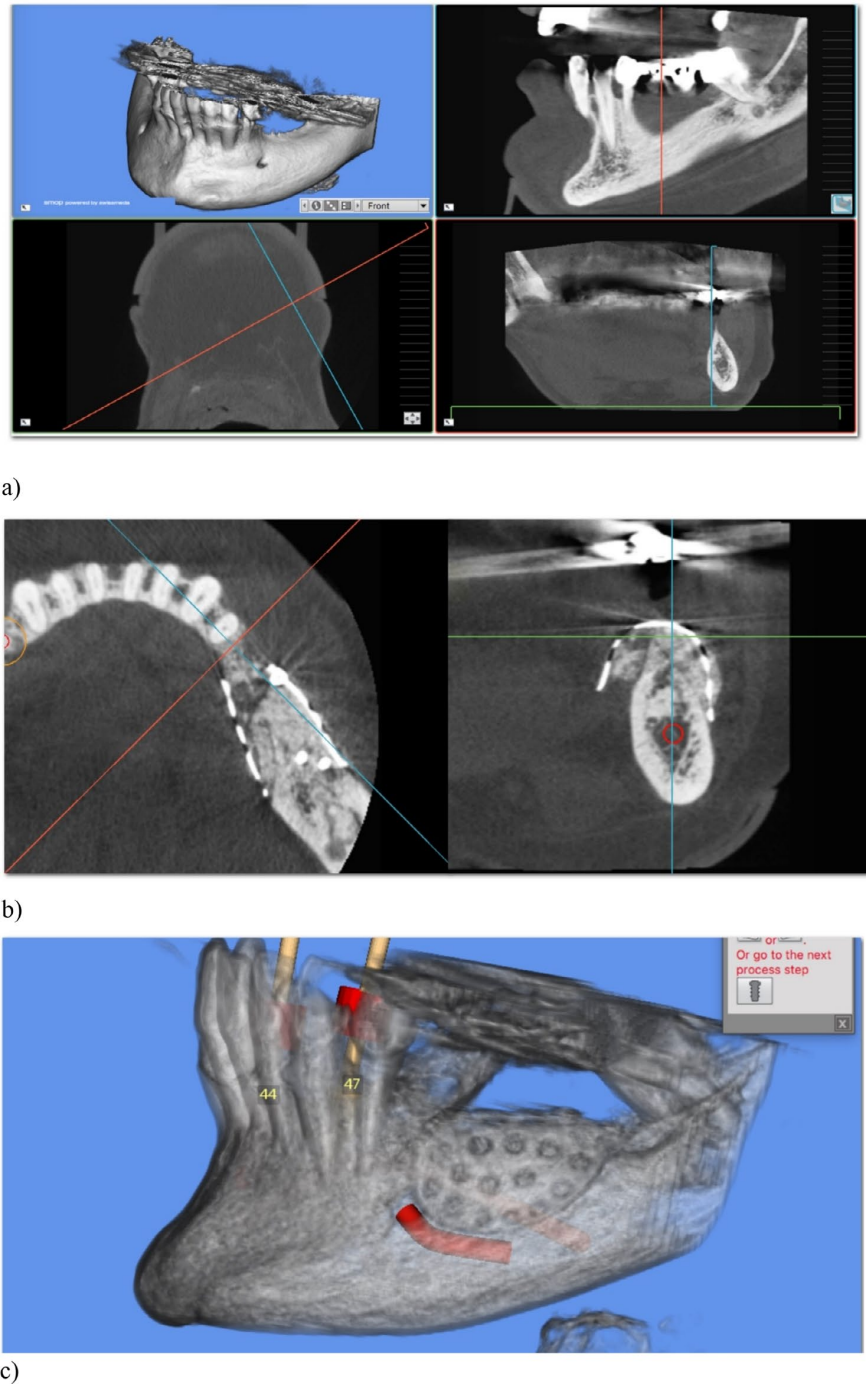
In figure (a), the sagittal and coronal planes are analyzed to study bone levels and density. In figure (b), the planes show the placement of the titanium mesh, (c) 3D reconstruction with the mesh on site.

Three‐dimensional CBCT images were obtained for each case and used both for preoperative planning and for the design of the patient‐specific titanium mesh. Digital datasets were employed to virtually plan graft volume, mesh morphology, and surgical execution (Figure 7).

In all cases dental implants were placed from 6 to 8 months after the bone augmentation procedure.

### 2.8 Implant Placement

The implants (Zimmer Biomet Certain implants), were inserted at the bone level following the instructions provided by the manifacturer. This implant system has a DCD surface (named Osseotite) and an internal hexagon connection.

For the purpose of this investigations implants with Xrays at the time of the prosthetic delivery and then 5 years after loading were included, in order to evaluate the marginal bone loss.

At all the steps standardized intraoral radiographs were obtained with a parallel technique, using a personalized film holder with the customization of the bite.

### 2.9 Statistical Analysis

Statistical analyses were performed to compare outcomes between the two treatment groups. Continuous variables were expressed as mean ± standard deviation. Intergroup comparisons of dimensional bone gain were conducted using an independent‐samples *t*‐test.

Given the observed difference in baseline age between the two groups, an adjusted analysis was performed to account for this potential confounding factor. An analysis of covariance (ANCOVA) was conducted for the primary outcomes (bone height and bone width at the 6‐month follow‐up), with treatment group included as the fixed factor and patient age entered as a covariate. This approach was adopted to assess whether age influenced the treatment effect and to obtain age‐adjusted intergroup comparisons.

Categorical variables, including the incidence of postoperative complications and the distribution of pseudo‐periosteum extent, were analyzed using Fisher's exact test due to the limited sample size.

All statistical tests were two‐tailed, and the level of significance was set at *α* = 0.05.

Post hoc power analysis was conducted to assess the ability of the study to detect intergroup differences. Cohen's *d* was calculated for the main outcomes, including bone height and width at 6‐month follow‐up (T1) for both maxillary and mandibular sites, to quantify the observed effect sizes and support interpretation of the resultsStatistical analyses were performed using SPSS version 29.0 (IBM Corp., Armonk, NY, USA).

## 3 Results

A total of 40 patients (24 males, 16 females) were included. Mean age was 40.7 years in the Tent‐pole group and 48.6 years in the Ti‐mesh group. ANCOVA adjusted for baseline age differences showed that age was not a significant covariate for bone height or width at 6 months (*p* > 0.05). No statistically significant differences were observed between groups after adjustment.

Baseline three‐dimensional bone measurements for both maxilla and mandible are presented in Table 1. In the Tent‐pole group, maxillary bone height was 5.05 ± 1.07 mm (95% CI: 4.12–5.98; range: 3.98–6.12) and width 2.55 ± 0.80 mm (95% CI: 1.92–3.18; range: 1.70–3.10). Mandibular bone height was 6.07 ± 1.08 mm (95% CI: 5.14–7.00; range: 4.98–7.20) and width 1.96 ± 0.94 mm (95% CI: 1.12–2.80; range: 1.10–2.90). In the Ti‐mesh group, maxillary bone height was 5.15 ± 1.15 mm (95% CI: 4.18–6.12; range: 4.00–6.39) and width 3.05 ± 1.02 mm (95% CI: 2.20–3.90; range: 2.00–4.10). Mandibular bone height was 6.15 ± 1.05 mm (95% CI: 5.22–7.08; range: 5.00–7.30) and width 2.05 ± 0.92 mm (95% CI: 1.21–2.89; range: 1.20–3.00).

**TABLE 1:** Patients evaluation for the procedure overall.

Question	Evaluation from 1 to 10
1‐How do you rate the duration of the surgery?	evaluation
2‐Did you feel any pain/discomfort during the procedure?	evaluation
3‐Please rate the pain experienced during the days after the surgery	evaluation
4‐Would you undergo again the same procedure?	evaluation

All augmented sites allowed successful implant placement. Summary statistics including mean, SD, 95% CI, and range are provided in Table 2.

**TABLE 2:** Descriptive statistics (mean ± SD, 95% CI, range) of baseline bone measurements by group and jaw.

Group	Jaw	Height (mm) Mean ± SD	95% CI	Range	Width (mm) Mean ± SD	95% CI	Range
Tent‐pole	Maxilla	5.05 ± 1.07	4.12–5.98	3.98–6.12	2.55 ± 0.80	1.92–3.18	1.70–3.10
Tent‐pole	Mandible	6.07 ± 1.08	5.14–7.00	4.98–7.20	1.96 ± 0.94	1.12–2.80	1.10–2.90
Ti‐mesh	Maxilla	5.15 ± 1.15	4.18–6.12	4.00–6.39	3.05 ± 1.02	2.20–3.90	2.00–4.10
Ti‐mesh	Mandible	6.15 ± 1.05	5.22–7.08	5.00–7.30	2.05 ± 0.92	1.21–2.89	1.20–3.00

The mean operative time was 72.7 min for Group A (Tenting‐pole) (range: 60–85) and 62.4 min for Group B (Ti‐mesh) (range: 60–65), as recorded prospectively during the procedures. These values reflect the actual intraoperative times observed in our cohort and are consistent with the surgical protocols applied. Differences with previously reported literature times may be due to variations in defect complexity, operator experience, and the specific surgical workflow used in this study.

### 3.1 Bone Augmentation

Three‐dimensional (3D) evaluations were performed at 6 months (T1) and compared with baseline (T0). At T0, Tent‐pole group patients had a mean maxillary bone height of 5.05 ± 1.07 mm (95% CI: 4.12–5.98; range: 3.98–6.12) and width of 2.55 ± 0.80 mm (95% CI: 1.92–3.18; range: 1.70–3.10), while mandibular height was 6.07 ± 1.08 mm (95% CI: 5.14–7.00; range: 4.98–7.20) and width 1.96 ± 0.94 mm (95% CI: 1.12–2.80; range: 1.10–2.90). In the Ti‐mesh group, maxillary height was 5.15 ± 1.15 mm (95% CI: 4.18–6.12; range: 4.00–6.39) and width 3.05 ± 1.02 mm (95% CI: 2.20–3.90; range: 2.00–4.10), while mandibular height was 6.15 ± 1.05 mm (95% CI: 5.22–7.08; range: 5.00–7.30) and width 2.05 ± 0.92 mm (95% CI: 1.21–2.89; range: 1.20–3.00).

At 6 months, Tent‐pole patients showed maxillary height of 8.70 ± 2.60 mm (95% CI: 7.22–10.18; range: 6.12–11.20) and width 4.70 ± 1.45 mm (95% CI: 3.63–5.77; range: 3.10–6.50), mandibular height 9.35 ± 2.32 mm (95% CI: 7.97–10.73; range: 7.20–12.00) and width 4.90 ± 1.34 mm (95% CI: 3.84–5.96; range: 2.90–6.20). Ti‐mesh patients exhibited maxillary height of 8.93 ± 2.54 mm (95% CI: 7.51–10.35; range: 6.39–12.10) and width 5.27 ± 1.54 mm (95% CI: 4.05–6.49; range: 4.10–7.00), mandibular height 9.25 ± 2.26 mm (95% CI: 7.88–10.62; range: 5.00–12.00) and width 5.30 ± 1.42 mm (95% CI: 4.12–6.48; range: 3.00–7.00); data are reported in Table 3.

**TABLE 3:** Six‐month (T1) three‐dimensional alveolar bone measurements in Tent‐pole and Ti‐mesh groups.

Group	Jaw	Height (mm) Mean ± SD	95% CI	Range	Width (mm) Mean ± SD	95% CI	Range
Tent‐pole	Maxilla	8.70 ± 2.60	7.22–10.18	6.12–11.20	4.70 ± 1.45	3.63–5.77	3.10–6.50
Tent‐pole	Mandible	9.35 ± 2.32	7.97–10.73	7.20–12.00	4.90 ± 1.34	3.84–5.96	2.90–6.20
Ti‐mesh	Maxilla	8.93 ± 2.54	7.51–10.35	6.39–12.10	5.27 ± 1.54	4.05–6.49	4.10–7.00
Ti‐mesh	Mandible	9.25 ± 2.26	7.88–10.62	5.00–12.00	5.30 ± 1.42	4.12–6.48	3.00–7.00

*Note:* Data are reported as mean ± SD, with 95% confidence intervals (CI) and range (minimum–maximum) for maxillary and mandibular sites.

Comparisons between groups were performed using Welch's *t*‐test for independent samples to account for potential unequal variances between groups. This approach is equivalent to a standard Student's *t*‐test when variances are equal but provides more robust results when variances differ.

**FIGURE 8:**
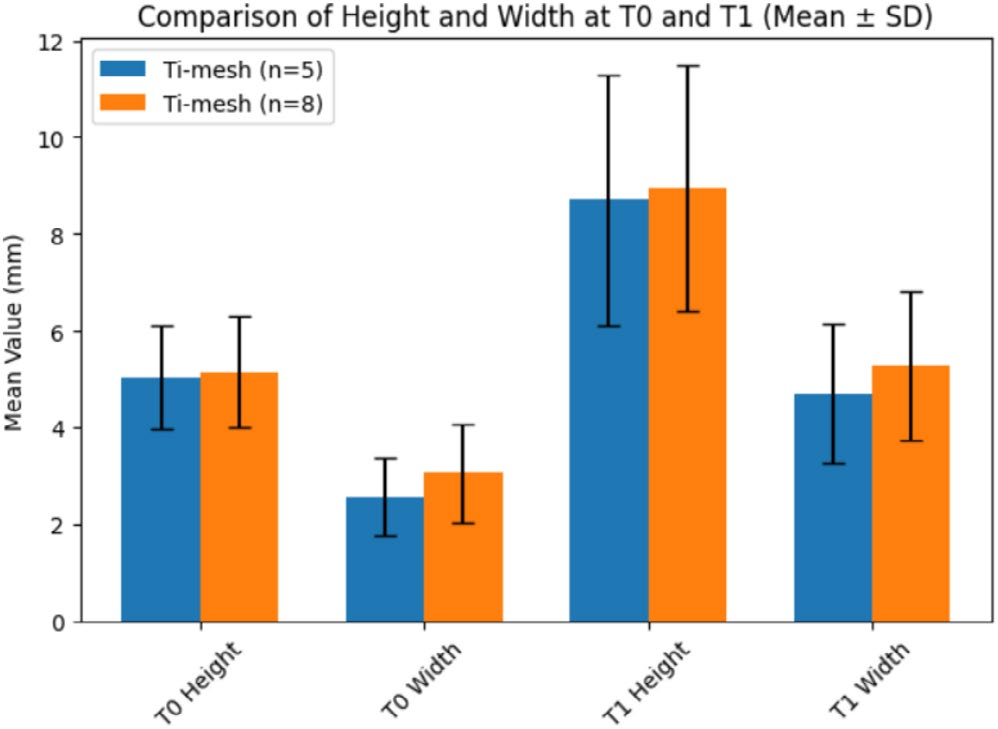
Bar chart showing the comparison of vertical (height) and horizontal (width) dimensions at baseline (T0) and at follow‐up (T1) between the two Ti‐mesh groups (*n* = 5 and *n* = 8). Data are presented as mean ± standard deviation (SD). No statistically significant differences were observed between groups at any time point (Welch's *t*‐test, *p* > 0.05).

**FIGURE 9:**
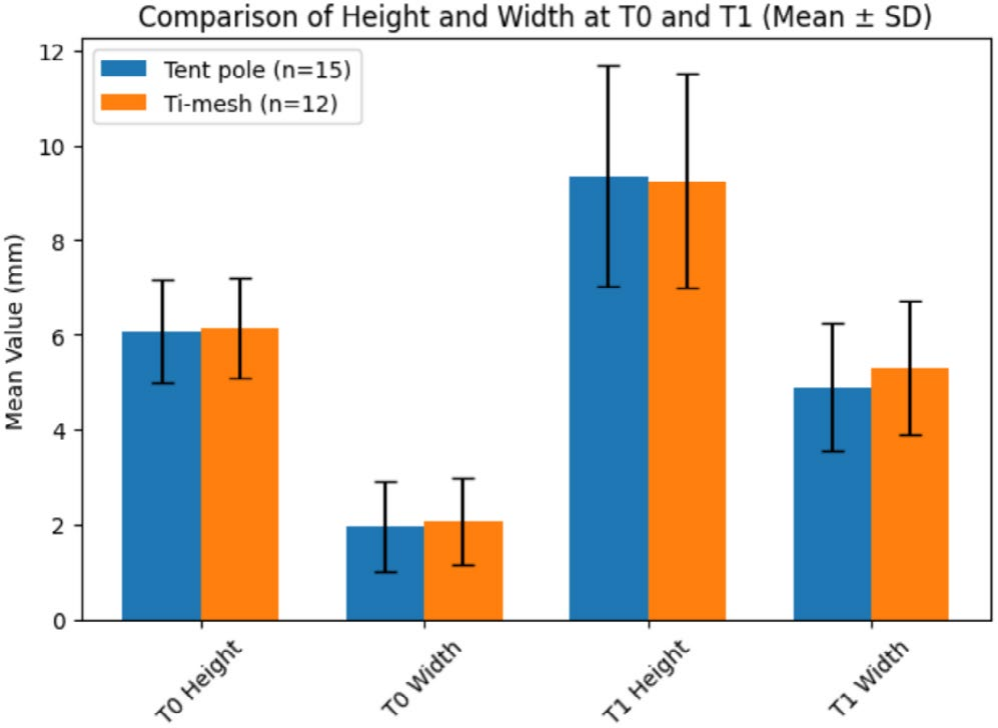
Bar chart comparing vertical (height) and horizontal (width) dimensions at baseline (T0) and follow‐up (T1) between the Tent pole group (*n* = 15) and the Ti‐mesh group (*n* = 12). Data are presented as mean ± standard deviation (SD). No statistically significant differences were observed between groups at any time point (Welch's *t*‐test, *p* > 0.05).

In Tables 4 and 5 and Figures 8 and 9 are reported intergroup comparisons of bone height and width at T0 and T1 using Welch's *t*‐test for independent samples.

**TABLE 4:** Comparison of maxillary bone height and width between Tent‐pole and Ti‐mesh groups at baseline (T0) and 6 months (T1) using Welch's *t*‐test for independent samples.

Timepoint	Variable	Tent pole group (*n* = 5) Mean ± SD	Ti‐mesh group (*n* = 8) Mean ± SD	Welch's t	*p*
T0	Height (mm)	5.05 ± 1.07	5.15 ± 1.15	−0.15	0.88
T0	Width (mm)	2.55 ± 0.80	3.05 ± 1.02	−0.93	0.37
T1	Height (mm)	8.70 ± 2.60	8.93 ± 2.54	−0.17	0.87
T1	Width (mm)	4.70 ± 1.45	5.27 ± 1.54	−0.69	0.51

*Note:* Data are reported as mean ± SD with *t*‐values and corresponding *p*‐values.

**TABLE 5:** Comparison of mandibular bone height and width between Tent‐pole and Ti‐mesh groups at baseline (T0) and 6 months (T1) using Welch's *t*‐test for independent samples.

Timepoint	Variable	Tent pole group(*n* = 15) Mean ± SD	Ti‐mesh group (*n* = 12) Mean ± SD	Welch's t	*p*
T0	Height (mm)	6.07 ± 1.08	6.15 ± 1.05	−0.19	0.85
T0	Width (mm)	1.96 ± 0.94	2.05 ± 0.92	−0.25	0.81
T1	Height (mm)	9.35 ± 2.32	9.25 ± 2.26	0.11	0.91
T1	Width (mm)	4.90 ± 1.34	5.30 ± 1.42	−0.75	0.46

*Note:* Data are reported as mean ± SD with *t*‐values and corresponding *p*‐values.

Welch's *t*‐test revealed no statistically significant differences between Tent‐pole and Ti‐mesh groups in maxillary or mandibular bone height and width at baseline (T0) or at the 6‐month follow‐up (T1) (*p* > 0.05 for all comparisons).

### 3.2 Marginal Bone Loss Around Implants

**FIGURE 10:**
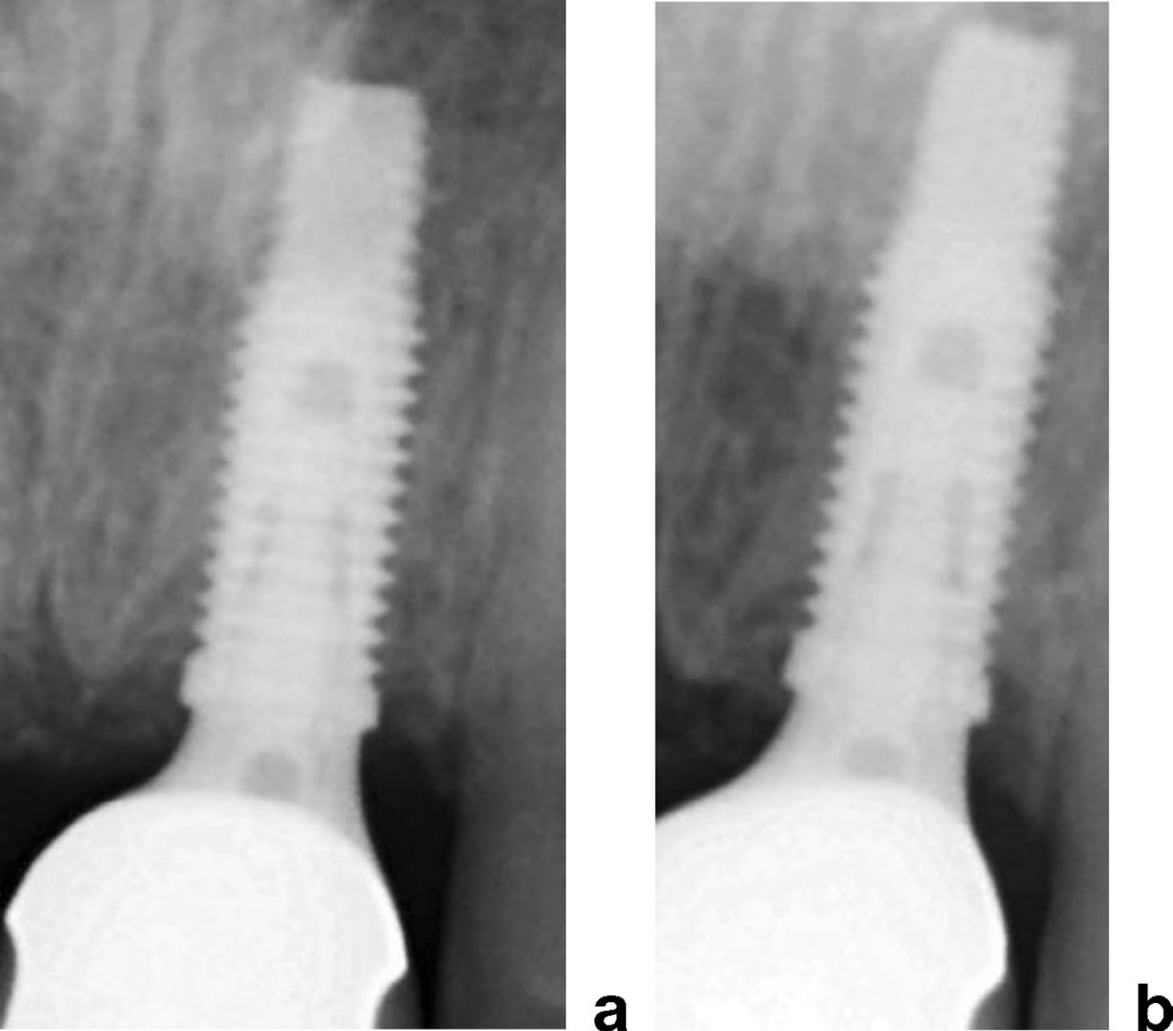
Case of Tent‐pole group reported in Figure 2 (a) bone levels at the prosthetic delivery, (b) bone levels 5 years after prosthetic delivery.

**FIGURE 11:**
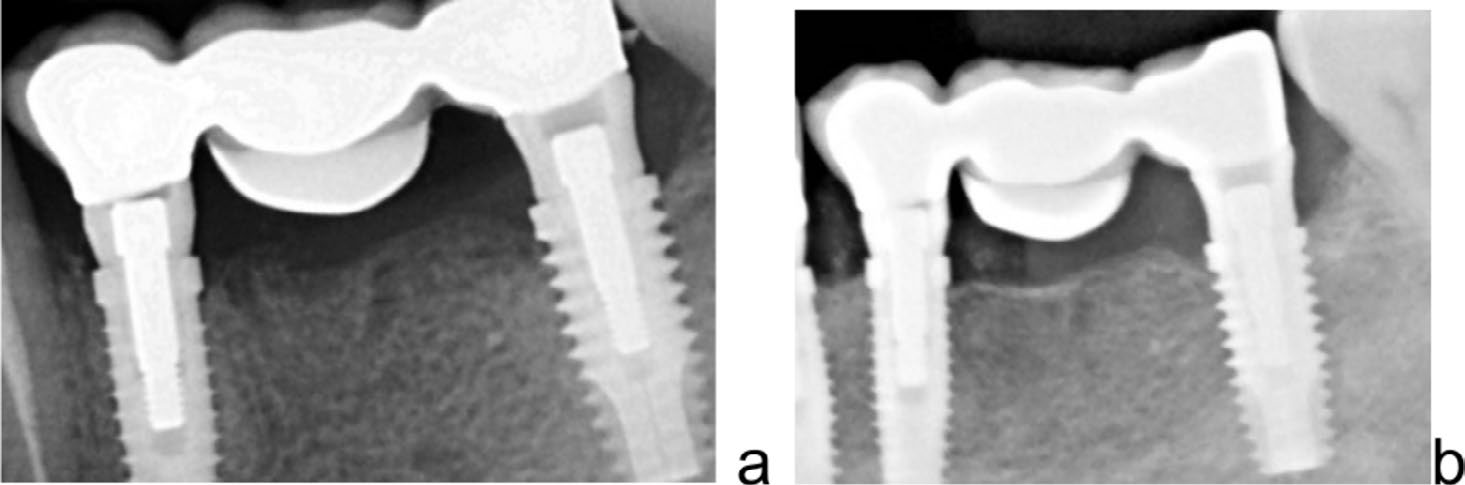
Case of Ti mesh group reported in Figures 3 and 4 (a) bone levels at the prostetich delivery, (b) bone levels 5 years after prosthetic delivery.

A total of 60 implants were placed in the 40 treated sites, with no implant failures. Bone resorption levels were evaluated at the time of prosthetic loading (post‐loading) and at 5 years post‐loading. At post‐loading, mean marginal bone loss (BL) was 1.37 ± 0.29 mm in the maxilla and 1.12 ± 0.19 mm in the mandible (95% CI: 1.22–1.52 and 1.03–1.21, respectively), showing a statistically significant difference between jaws (*p* = 0.0015). At 5 years post‐loading, mean BL increased to 1.80 ± 0.38 mm in the maxilla and 1.35 ± 0.27 mm in the mandible (95% CI: 1.57–2.03 and 1.19–1.51, respectively; *p* < 0.0001) (Figures 10 and 11).

When comparing anterior implants only, no statistically significant differences were observed between upper and lower jaw at either time point. Posterior implants exhibited greater bone loss than anterior ones, with upper posterior elements showing 1.92 ± 0.35 mm (95% CI: 1.70–2.14) and lower posterior elements 1.26 ± 0.22 mm (95% CI: 1.10–1.42) at 5 years post‐loading (*p* < 0.0001). No significant differences were observed at post‐loading (upper posterior 1.42 ± 0.22 mm vs. lower posterior 1.07 ± 0.20 mm; *p* < 0.0001). These data confirm stable bone levels over time and successful osseointegration of all 60 implants and are reported in Table 6.

**TABLE 6:** Bone Level at post loading and 5 years after stratified according to the position of the implants.

Jaw/Position	Timepoint	BL (mm) Mean ± SD	95% CI	Range
Maxilla (all)	Post‐loading	1.37 ± 0.29	1.22–1.52	1.0–1.8
Mandible (all)	Post‐loading	1.12 ± 0.19	1.03–1.21	0.9–1.5
Maxilla (all)	5 years	1.80 ± 0.38	1.57–2.03	1.3–2.5
Mandible (all)	5 years	1.35 ± 0.27	1.19–1.51	1.0–1.8
Maxillary anterior	Post‐loading	1.30 ± 0.25	1.12–1.48	1.0–1.7
Mandibular anterior	Post‐loading	1.08 ± 0.20	0.95–1.21	0.9–1.5
Maxillary anterior	5 years	1.72 ± 0.35	1.50–1.94	1.2–2.3
Mandibular anterior	5 years	1.28 ± 0.22	1.12–1.44	1.0–1.6
Maxillary posterior	Post‐loading	1.42 ± 0.22	1.26–1.58	1.1–1.8
Mandibular posterior	Post‐loading	1.07 ± 0.20	0.93–1.21	0.9–1.4
Maxillary posterior	5 years	1.92 ± 0.35	1.70–2.14	1.4–2.5
Mandibular posterior	5 years	1.26 ± 0.22	1.10–1.42	1.0–1.6

### 3.3 Complications

Pseudoperiosteum formation was evaluated at 6 months post‐surgery and classified according to a three‐tier system: Class 0 = none, Class 1 = < 1 mm, Class 2 = 1–2 mm, Class 3 = > 2 mm [29]. In the Tent‐pole group (Group A), five mandibular implants showed Class 2 pseudoperiosteum, with no cases in the maxilla. In the Ti‐mesh group (Group B), two implants presented Class 2 pseudoperiosteum, one in the maxilla and one in the mandible. No Class 3 cases were observed in either group. Fisher's Exact Test showed no statistically significant difference in pseudoperiosteum occurrence between groups (*p* = 0.13). No major complications, including infections, were observed in either group.

### 3.4 Patients' Satisfaction Score

All patients completed the questionnaire described in the Materials & Methods section. For each item, patients assigned a score from 1 (lowest) to 10 (highest) according to their experience. The values reported in Table 7 represent the mean scores for each question in each group. No formal statistical comparison was performed, as the aim of this analysis was purely descriptive, to provide an overview of patient‐reported experience. Results indicate similar perceptions between groups.

**TABLE 7:** Mean scores of patient responses to the questionnaire (1 = lowest satisfaction/experience, 10 = highest) in Tent‐pole and Ti‐mesh groups.

Question	Tent‐pole group	Ti‐mesh group
1‐How do you rate the duration of the surgery?	4	3
2‐Did you feel any pain/discomfort during the procedure?	1	1
3‐Please rate the pain experienced during the days after the surgery	4	3
4‐would you undergo again the same procedure?	3	4

*Note:* No formal statistical comparisons were performed; values are descriptive and reflect the average perception of patients in each group.

### 3.5 Intraoperative Timing

Intraoperative time was recorded for all surgical procedures, defined as the interval from the initial incision to the placement of the final suture. The mean operative time was 72.7 min (range: 60–85) for the Tent‐pole group and 62.4 min (range: 60–65) for the Ti‐mesh group. These times were prospectively recorded during surgery and independently verified by the operating surgeon, accurately reflecting the actual duration required for each procedure. Statistical analysis indicated a shorter operative time for the Ti‐mesh group (*p* < 0.05). Differences from literature‐reported times may be due to variations in defect complexity, surgical technique, and operator workflow.

## 4 Discussion

The present retrospective study compared two guided bone regeneration (GBR) protocols—resorbable collagen membranes supported by tenting screws and customized CAD/CAM titanium meshes—in the treatment of horizontal and/or vertical alveolar ridge defects. Within the limits of the study design, both approaches resulted in substantial and clinically relevant ridge augmentation, with no statistically significant differences in overall dimensional bone gain after 6 months. These findings highlight that the previously reported post hoc power estimate, based on a large effect size (Cohen's *d* = 0.91), overestimated the study's ability to detect differences. The observed small effect sizes indicate that the study was underpowered to detect subtle intergroup differences, which should be considered when interpreting the results.

Both treatment modalities achieved mean vertical and horizontal gains in the range of approximately 3.5–4.5 mm, in line with previously published data [30]. Similar magnitudes of bone regeneration have been reported for GBR procedures performed using either barrier membranes or titanium meshes [31, 32, 33]. In particular, prior studies have documented mean vertical gains of approximately 4.0–4.5 mm using non‐resorbable membranes and titanium meshes, as well as comparable horizontal gains following GBR with collagen membranes or mesh‐based techniques [34]. These findings indicate that, when GBR principles are correctly applied, both approaches are capable of producing predictable and clinically meaningful bone regeneration [35]. The present results therefore confirm that neither technique is inherently superior in terms of short‐term volumetric bone gain under standard clinical conditions.

Differences between the two approaches may emerge when space maintenance and volume stability are considered. Titanium meshes provide greater intrinsic rigidity compared with resorbable membranes, which may translate into improved resistance to soft‐tissue pressure, particularly in non‐contained or vertically demanding defects. In the present study, both groups demonstrated stable graft volumes at 6 months; however, mesh‐treated sites showed a non‐significant trend toward reduced volumetric collapse. This observation is consistent with recent evidence suggesting enhanced volume preservation with mesh‐based GBR [36]. Li et al. [37] reported a higher percentage of graft volume maintenance over time with titanium meshes, particularly at the buccal aspect, and meta‐analyses have indicated lower resorption rates for meshes (approximately 23%–25%) compared with membrane‐based techniques (approximately 35%). Although such differences may not always reach statistical significance in small samples, they may be clinically relevant in esthetically sensitive areas or when precise ridge contours are required.

Conversely, GBR procedures using resorbable membranes rely on adjunctive fixation devices, such as tenting screws, to achieve adequate space maintenance [38, 39]. When appropriately applied, this strategy has been shown to significantly enhance regenerative outcomes. In the present study, the use of tenting screws allowed the membrane‐based protocol to achieve bone gains comparable to those obtained with titanium meshes, confirming the importance of mechanical support regardless of the barrier material. These findings support the concept that both approaches are effective when space maintenance, graft stability, and primary wound closure are adequately ensured.

At the 5‐year follow‐up, all 60 implants placed across the 40 treated sites remained successfully osseointegrated, with no implant failures reported in either group. Marginal bone levels were well preserved over time, demonstrating stable peri‐implant conditions. In the Tent‐pole group, mean marginal bone loss measured 1.82 ± 0.36 mm in the maxilla and 1.38 ± 0.28 mm in the mandible, while in the Ti‐mesh group, mean bone loss was 1.78 ± 0.40 mm in the maxilla and 1.32 ± 0.26 mm in the mandible. These findings indicate that, despite the differences in GBR approach, both techniques provided comparable long‐term support for peri‐implant bone maintenance [14, 40]. The absence of implant failures further confirms the clinical reliability of both protocols, highlighting that when GBR principles are correctly applied, favorable implant outcomes and durable marginal bone stability can be achieved regardless of the barrier material or fixation strategy [41, 42].

An additional finding of interest concerns the formation of a pseudo‐periosteum beneath barrier devices. Pseudo‐periosteum was observed in both groups, with 5 cases in the Tent‐pole group (all mandibular) and 2 cases in the Ti‐mesh group. No statistically significant differences were detected (Fisher's Exact Test, *p* = 0.13). Previous reports have described this fibrous connective tissue layer as exceeding 1 mm in thickness beneath rigid barriers and classified it as type 2 or type 3 pseudo‐periosteum [28]. The clinical relevance of this tissue remains debated. While it has been hypothesized that a thick fibrous layer could limit mineralized bone formation beneath the barrier, other authors have suggested that pseudo‐periosteum may act as a secondary protective layer, particularly in the event of late barrier exposure [16, 18, 43].

In the present study, no adverse clinical effects were associated with the presence of pseudo‐periosteum. These observations are in agreement with previous reports indicating that pseudo‐periosteum formation represents a benign biological response, whose clinical impact is largely dependent on effective soft‐tissue management and primary wound closure [44]. The absence of major complications such as infection or early exposure in the present cohort should be interpreted in light of the limited sample size and retrospective design. Reported complication rates for CAD/CAM titanium meshes and conventional GBR techniques vary widely in the literature, with randomized clinical trials and prospective studies reporting exposure rates ranging from approximately 10% to 30% [33, 45], often without compromising final bone regeneration outcomes. In this context, the lack of major complications observed in the present study is consistent with previously published data, particularly in cohorts treated with meticulous soft‐tissue management and careful case selection. Larger prospective and randomized studies are nonetheless required to better quantify complication risk and to directly compare different GBR techniques under controlled conditions.

Patient‐reported outcomes further support the clinical applicability of both GBR techniques. Overall patient satisfaction was high, and postoperative discomfort was limited, with no meaningful differences between the two groups. Postoperative pain, swelling, and analgesic consumption were low and comparable between membrane‐ and mesh‐based procedures. Mean scores indicated similar perceptions between groups; formal statistical testing was not performed as the questionnaire was descriptive. This finding is consistent with the limited available literature on patient‐reported outcome measures in GBR. For example, Cucchi et al. reported no significant differences in pain perception or quality‐of‐life indicators between patients treated with titanium meshes and those treated with non‐resorbable membranes [36, 46]. These data suggest that the requirement for a secondary mesh removal procedure does not necessarily translate into a worse patient experience when surgical protocols are carefully executed [47].

From a practical standpoint, each technique presents specific advantages and limitations. The collagen membrane with tenting screws represents a widely adopted and familiar approach, with the advantage of complete material resorption and avoidance of a second surgical procedure. However, it requires meticulous intraoperative handling, precise screw positioning, and careful membrane adaptation to prevent collapse, particularly in non‐contained defects [39, 42, 48, 49, 50]. In contrast, customized titanium meshes benefit from digital planning and CAD/CAM fabrication, allowing for passive adaptation, reduced intraoperative manipulation, and improved space maintenance [46, 51, 52]. The rigidity and predefined shape of the mesh facilitate preservation of the intended graft volume, particularly in vertically challenging reconstructions. These advantages must be balanced against the need for advanced preoperative planning, higher costs, and a second surgical procedure for mesh removal.

Clinically, both approaches allowed implant placement without the need for additional grafting procedures. Although not statistically significant, mesh‐treated sites in the present study often exhibited favorable ridge morphology and emergence profiles, suggesting a potential qualitative advantage in selected cases. While previous studies have reported a higher incidence of soft‐tissue dehiscence with titanium meshes compared with collagen membranes [53], exposures in the present cohort were infrequent and could be managed conservatively. Previous reports have similarly indicated that late mesh exposure does not necessarily compromise regenerative outcomes, particularly when a mature pseudo‐periosteum is present [15, 16].

The results of this study should be interpreted considering its limitations. The retrospective design and limited sample size restrict the ability to detect intergroup differences and preclude definitive causal conclusions. In addition, while the primary regenerative outcomes were assessed at the time of implant placement, allowing evaluation of early bone regeneration, long‐term follow‐up was limited to peri‐implant marginal bone level measurements at 5 years. Although this provides meaningful information on peri‐implant stability, it does not allow a comprehensive assessment of long‐term volumetric remodeling of the regenerated bone or broader implant survival outcomes. Future prospective studies with larger cohorts, longer follow‐up, and standardized volumetric assessments are needed to further elucidate potential differences between these GBR techniques.

Moreover the clinical and radiographic outcomes, intraoperative timing represents a relevant practical parameter when comparing GBR techniques. The mean operative time was 72.7 min for the Tent‐pole group and 62.4 min for the Ti‐mesh group (*p* < 0.05). All times were prospectively recorded and independently verified. This difference can likely be attributed to the passive fit and predefined geometry of CAD/CAM‐fabricated meshes, which reduce the need for intraoperative trimming, bending, and repeated adjustments. Conversely, the membrane‐based protocol requires meticulous adaptation of the membrane and precise placement of tenting screws to ensure adequate space maintenance, potentially increasing surgical complexity and duration. Although operative time does not directly affect regenerative outcomes, shorter procedures may reduce surgical stress for both the patient and the operator and improve overall workflow efficiency, particularly in complex or multi‐site augmentations.

The present study has several strengths and limitations that should be acknowledged. Among its strengths, this retrospective analysis reflects real‐world clinical practice and includes standardized surgical protocols, consistent outcome assessment, and a long‐term follow‐up evaluating marginal bone level changes up to 5 years, with no implant failures observed. In addition, quantitative radiographic measurements allowed for an objective comparison between the two GBR techniques.

However, some limitations must be considered. First, the retrospective design inherently limits control over data collection and documentation, as the study was not originally conceived as a prospective clinical trial. Second, the relatively limited sample size may reduce the statistical power to detect small differences between groups and may partially explain the absence of major complications. Third, not all clinical cases were documented with complete photographic records suitable for publication, particularly for re‐entry procedures and vertical augmentations, which restricted the number of illustrative clinical examples. Finally, the absence of randomization and blinding may introduce selection bias, and the results should therefore be interpreted with caution. Future prospective and randomized clinical trials with larger cohorts are warranted to further validate these findings.

In conclusion, both customized CAD/CAM titanium meshes and resorbable collagen membranes supported by tenting screws proved effective for alveolar ridge augmentation in compromised sites. While overall bone gain was comparable, customized meshes demonstrated a tendency toward improved space maintenance and contour control in more demanding defect configurations, whereas membrane‐based GBR offered a simpler and fully resorbable alternative when adequate mechanical support was provided. The selection of the GBR technique should therefore be guided by defect morphology, clinical objectives, and surgical expertise rather than expectations of superior bone gain alone.

## 5 Conclusions

Within the limitations of this retrospective study, both customized CAD/CAM titanium meshes and resorbable collagen membranes supported by tenting screws proved to be effective approaches for guided bone regeneration in horizontal and/or vertical alveolar ridge defects. Comparable dimensional bone gains in terms of ridge height and width were achieved with both techniques, allowing predictable implant placement without the need for additional augmentation procedures.

No statistically significant differences in linear bone gain were observed between the two groups. Therefore, based on the outcomes measured in the present study, neither approach demonstrated a clear quantitative advantage over the other in terms of regenerated bone dimensions.

From a clinical perspective, customized titanium meshes may represent a useful option in complex defect scenarios where rigid support is required, while membrane‐based GBR offers a simpler and fully resorbable alternative when adequate mechanical stabilization can be achieved. However, these considerations should be interpreted as clinical observations rather than as demonstrated quantitative advantages.

Overall, the choice of the GBR technique should be primarily guided by defect morphology, surgical planning, and clinical objectives rather than by expectations of superior bone gain. Further prospective studies incorporating volumetric analyses and larger cohorts are warranted to better characterize potential differences in space maintenance and long‐term outcomes between these approaches.

## Author Contributions

Concept/Design: Wurtz G. and De Angelis N. Data analysis/interpretation: De Angelis N., Menini M., and Pesce P. Drafting article: Wurtz G. and Bagnasco F. Critical revision of article: Baldi D. Approval of article: De Angelis N., Menini M., and Pesce P. Statistics: Wurtz G. and De Angelis N. Data collection: Wurtz G.

## Funding

The authors have nothing to report.

## Conflicts of Interest

The authors declare no conflicts of interest.

## Data Availability

The data that support the findings of this study are available on request from the corresponding author. The data are not publicly available due to privacy or ethical restrictions.
